# Education Research: Changes in Medical Students' Knowledge and Attitudes Toward Clinical Death After Teaching the Philosophy of Death

**DOI:** 10.1212/NE9.0000000000200055

**Published:** 2023-03-23

**Authors:** Nicholas Ludka, Abram Brummett, Jason Adam Wasserman

**Affiliations:** From the Department of Foundational Medical Studies, Oakland University William Beaumont School of Medicine, Rochester, MI.

## Abstract

**Background and Objectives:**

Varied meanings of death within medicine, bioethics, and society at large often produce disagreement and frustration between physicians and surrogate decision makers. We investigated whether teaching medical students about the philosophical aspects of death would change their attitude toward surrogate decision makers who assert nonstandard views of death.

**Methods:**

An 80-minute lecture covering philosophical debates surrounding medico-legal standards of death was given to second-year medical students at Oakland University William Beaumont School of Medicine during a neuroscience course. Participants completed a questionnaire containing Likert scale and open-ended questions before and after the intervention assessing their acceptance of, frustration toward, and likelihood of accommodating a request for surrogate decision makers who posited either a whole-brain, high-brain, or circulatory view of death. Change in knowledge was analyzed using the McNemar test, whereas attitudinal scores were compared with paired *t* tests. Open-ended responses were narratively analyzed to identify themes that elaborate quantitative findings.

**Results:**

A total of 43 paired responses were analyzed from second-year medical students. Following the intervention, students expressed less frustration (${\bar{\text{χ}}}_{\text{diff}}$ = −0.64, 95% CI −0.15 to −1.15), greater likelihood of accommodating ventilator removal (${\bar{\text{χ}}}_{\text{diff}}$ = 0.60, 95% CI 0.41–0.85), and greater acceptance (${\bar{\text{χ}}}_{\text{diff}}$ = 0.63, 95% CI 0.28–0.91) of surrogates who endorsed a whole-brain view of death. Although students rated the high-brain view as more acceptable after the lecture (${\bar{\text{χ}}}_{\text{diff}}$ = 0.63, 95% CI 0.28–0.91), they were not more likely to remove a ventilator from a patient who had experienced high-brain death (${\bar{\text{χ}}}_{\text{diff}}$ = 0.19, 95% CI −0.30 to 0.67). Students were less likely to continue artificial ventilation for a brain-dead patient (${\bar{\text{χ}}}_{\text{diff}}$ = −0.61, 95% CI −0.91 to −0.30) despite no change in frustration toward the surrogate (${\bar{\text{χ}}}_{\text{diff}}$ = −0.26, 95% CI 0.20 to −0.70).

**Discussion:**

Changes in attitudes across the 3 views of death suggest that increased awareness of the philosophical debate facilitates reflection of students' understanding and opinion of death. These findings support implementation of educational interventions to prepare students for future work with surrogate decision makers holding diverse sets of views on death.

The Uniform Determination of Death Act (UDDA) established a common legal definition of death in 1981. The UDDA states that a person is dead if (1) there is irreversible cessation of circulatory and respiratory function or (2) there is irreversible cessation of all functions of the entire brain, including the brain stem.^1^ The latter criterion is known as whole-brain death (WBD). This definition has been adopted by all 50 states, with only small variation present in 12 states.^2^ However, this near-universal adoption should not be mistaken as universal agreement. There is considerable debate within medicine and bioethics regarding the legitimacy of other views of death, namely high-brain death (HBD) and circulatory death (CD). The HBD view posits irreversible cessation of higher brain functioning, namely, capacity for consciousness, as sufficient evidence for declaring death.^3^ Others argue for a CD view where death requires irreversible cessation of circulation. Importantly, on this view, patients with WBD with preserved cardiac function would be considered alive. The circulatory view was famously thrust into the public spotlight with the case of Jahi McMath, who was declared WBD, but whose family objected that she was alive because her heart was still beating.^4^

Previous studies have shown knowledge deficits among medical students about WBD. A study on the effect of brain death education at New York University School of Medicine found that students had a median score of 53% on a comprehensive questionnaire on the medical and legal criteria for WBD.^5^ In a similar study, only 33% of medical students at New Mexico School of Medicine scored a perfect score on a 5-question survey on the basic physiologic findings in WBD.^6^ Similar misunderstandings surrounding basic facts about WBD have been reported elsewhere.^7-10^ This likely owes to a lack of education on medical and legal criteria for death determination within medical education.

Although WBD testing is most commonly performed by neurologists, neurosurgeons, or intensivists, there is widespread variation in hospital policy in who can perform it. In fact, more than 50% of hospitals do not require a neurologist or neurosurgeon to perform testing,^11^ suggesting that there is a high likelihood that medical students will be placed into situations during rotations, residency, or beyond where they will need to have baseline knowledge about WBD. This has motivated a call for widespread educational initiatives that better prepare medical professionals to diagnose and discuss WBD.^12,13^

Furthermore, although there is a growing literature on medical students' knowledge about WBD, there is scant evidence related to students' attitudes toward different views of death. These have only been studied indirectly in the context of attitudes toward organ transplantation from WBD donors, which is widely accepted by a majority of medical students.^14,15^ This is troubling because the varying views of death represented by different stakeholders in the clinical setting can lead to deep-seated disagreements about the plan of care. A working knowledge of different views of death is therefore a form of, or at least akin to, cultural competency and is critical for mitigating such disagreements. We sought to understand student attitudes toward WBD, HBD, and CD, while also addressing gaps in students' knowledge of these views, by developing an educational intervention on the history, application, and philosophical debate surrounding the medico-legal standard of death. Changes in students' attitudes toward WBD, HBD, and CD were investigated to understand how a survey of these complex issues may facilitate reflection on, and alteration to, their attitudes toward the 3 views of death.

## Methods

### Educational Intervention

The sample was drawn from students at Oakland University William Beaumont School of Medicine, an allopathic medical school in the Midwest who participated in a 4-week neuroscience course in the middle of their second year. A mandatory 80-minute lecture on the history of brain death, the current medical and legal standards for death as described by the UDDA, and nonstandard views of death was delivered in the final week of that course. The lecture included several clinical cases that required students to apply the conceptual material.

### Survey Design

The primary objective of determining student's attitude change was achieved through cases involving a surrogate decision maker who asserted a WBD, HBD, or CD view of death. The vignettes were written with a clear description of the mechanism of injury and resulting functional status of the patient to focus on the view of death and less on understanding of acute brain injury or prognostication in the setting of acute brain injury. Special attention was paid in writing the vignettes to prevent biasing factors like the patient's age, sex, and mechanism of injury (see eAppendix 1, links.lww.com/NE9/A22). In each case, students' attitudes toward the surrogate's view of death were measured on a 6-point Likert scale. The survey items were written to capture 3 facets of attitude: affect, behavior, and cognition.^16^ The affective measure asked students to rate their level of frustration toward the surrogate; the behavioral measure asked students their likelihood of accommodating a request to remove life support in the WBD and HBD scenarios or continue life support in the CD scenario; and the cognitive measure asked students to rate the acceptability of the surrogate's view of death. An optional open-ended response allowed students to elaborate on their responses. The secondary objective of determining student's knowledge of the current medico-legal standards of death was investigated using multiple choice questions that assessed accuracy of understanding of the UDDA and whether it could be appropriately applied to the 3 cases.

### Data Collection and Analysis

Students were asked to voluntarily complete the survey before and after attending the lecture. A recording of the lecture was made available to all students shortly after its delivery to avoid bias toward students who attended in person. There was no control group for this study. The McNemar test was used to compare student knowledge of the medico-legal standards of death. Effect size for knowledge is reported as an odds ratio (OR). The mean score of the affective, behavioral, and cognitive measure was compared using a paired *t* test with the level of significance set a priori at α < 0.05. The 95% confidence intervals reported below represent the mean differences. Cohen *d* was used to measure the effect size for attitude-based measures. Nonpaired responses were also analyzed to provide further context to the paired data and compared with paired data to prevent systematic bias. All statistical tests were performed using SPSS Statistics 28.0.0.

Open-ended responses were analyzed first with an open coding strategy, after which core categories that reflected patterns of codes were identified. Finally, themes represented across these patterns were identified. Themes are described and exemplified by representative quotations below to give further clarity to the quantitative data.

### Standard Protocol Approvals, Registrations, and Patient Consents

The research satisfied category 2 exemption criteria (research involving educational tests) according to 45 CFR § 46.104(d)(2) and was granted exempt status by the Institutional Review Board at Oakland University (IRB-FY2022-173). An information sheet that described the purpose and procedure of the study was given at the beginning of both prelecture and postlecture surveys.

### Data Availability

Anonymized data not published within this article will be made available by request from any qualified investigator.

## Results

### Description of the Cohort

Among the 125 participants, 64 prelecture responses were collected, with 43 postlecture responses successfully paired for analysis. Across all measures, there were no differences in preintervention results between paired and nonpaired data. The class was 53% female and 47% male. Eighty percent of students were 20–24 years of age, and 20% were 25 years of age or older. The class was 57% White, 27% Asian, and 7% underrepresented in medicine, with 11% of students responding other.

### Baseline Knowledge and Attitudes Toward 3 Views of Death

Before the intervention, 48% of students could correctly identify the 2 criteria for determination of death contained in the UDDA (Figure 2A). When given a case of a surrogate who asserted a CD, HBD, or WBD view, 67%, 74%, and 84% of students correctly identified each view's consistency or inconsistency with the medico-legal standards, respectively (Figure 2B). Sixty-five percent of students understood that death could be determined despite the presence of a heartbeat, and 58% understood that a patient who had irreversibly lost consciousness, but continued all other bodily functions, was alive (Figure 2C).

Furthermore, prior to the intervention, students expressed less frustration (${\bar{\text{χ}}}_{\text{diff}}$ = −0.72, 95% CI −1.20 to −0.24, *d* = 0.46), a greater likelihood of accommodating the surrogate's request (${\bar{\text{χ}}}_{\text{diff}}$ = 1.21, 95% CI 0.91–1.51, *d* = 1.22), and viewed the WBD view as more acceptable (${\bar{\text{χ}}}_{\text{diff}}$ = 0.95, 95% CI 0.68–1.23, *d* = 1.06) compared with the HBD view. A similar result is seen when comparing the students' attitudes toward a WBD and CD view, where students expressed less frustration (${\bar{\text{χ}}}_{\text{diff}}$ = −1.28, 95% CI −0.66 to −1.90, *d* = 0.63), a higher likelihood of accommodating the WBD surrogate's request (${\bar{\text{χ}}}_{\text{diff}}$ = 1.80, 95% CI 1.23–2.35, *d* = 0.98), and viewed the WBD surrogate's view of death as more acceptable (${\bar{\text{χ}}}_{\text{diff}}$ = 1.47, 95% CI 1.00–1.93, *d* = 0.96). There was no difference between the HBD view and the CD view in students' frustration (${\bar{\text{χ}}}_{\text{diff}}$ = −0.56, 95% CI −1.12 to 0.06, *d* = 0.28), likelihood of accommodation (${\bar{\text{χ}}}_{\text{diff}}$ = 0.58, 95% CI −0.50 to 1.21, *d* = 0.28), or acceptability (${\bar{\text{χ}}}_{\text{diff}}$ = 0.51, 95% CI −0.02 to 1.05, *d* = 0.29).

**Figure 1:**
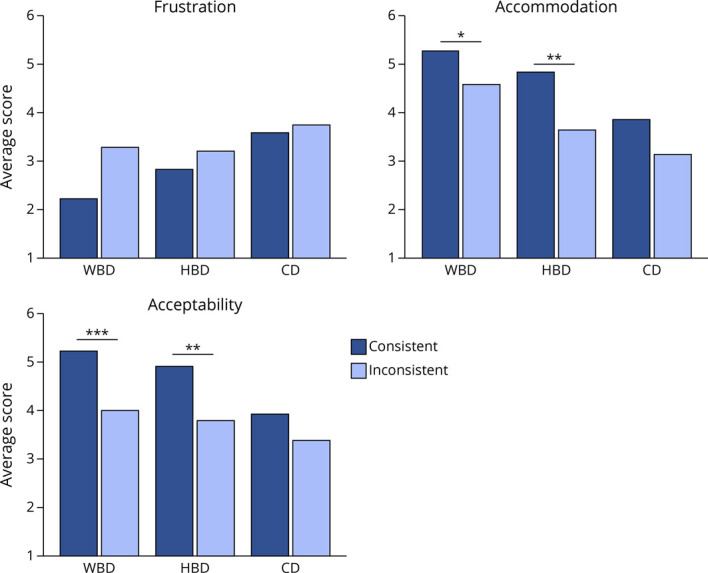
Relationship Between Medical Students' Attitudes and the Perceived Medico-Legal Standing of 3 Views of Death Preintervention average scores of the affective, behavioral, and cognitive measures of attitude for students who identified the WBD view as consistent (n = 36) or inconsistent (n = 7), the HBD view as consistent (n = 11) or inconsistent (n = 32), and the CD view as consistent (n = 14) or inconsistent (n = 29), with the current medico-legal standard. Responses were scored on a Likert scale from strongly disagree (1) to strongly agree (6). Statistics were calculated using an independent *t* test. *p < 0.05, **p < 0.01, and ***p < 0.001. CD = circulatory death; HBD = high-brain death; WBD = whole-brain death.

In the preintervention responses, students who identified the surrogate's whole-brain view of death as being consistent with the current medico-legal standards of death also had a higher likelihood of accommodating the request (${\bar{\text{χ}}}_{\text{diff}}$ = 0.71, 95% CI 0.006–1.41, *d* = 0.84) and rated the view of death as more acceptable (${\bar{\text{χ}}}_{\text{diff}}$ = 1.22, 95% CI 0.64–1.81, *d* = 1.74) than students who answered that the view is inconsistent with current standards (Figure 1). There was no difference in student's frustration toward WBD between those that thought it was either consistent or inconsistent with current medico-legal standards (${\bar{\text{χ}}}_{\text{diff}}$ = −1.06, 95% CI −2.20 to 0.07, *d* = 0.78). Similar results exist for students who incorrectly identified HBD and CD as consistent with medico-legal standards (Figure 1). That is, for each scenario, regardless of whether they were correct, students who believed that the surrogate's view of death was consistent with the medico-legal standard were more likely to accommodate that surrogate's request and rated that surrogate's view as more acceptable regardless of whether they were correct in their legal assessment. Student's legal assessment of each view was independent of their frustrations toward the view (Figure 1).

### Change in Knowledge of 3 Views of Death Following Educational Intervention

**Figure 2:**
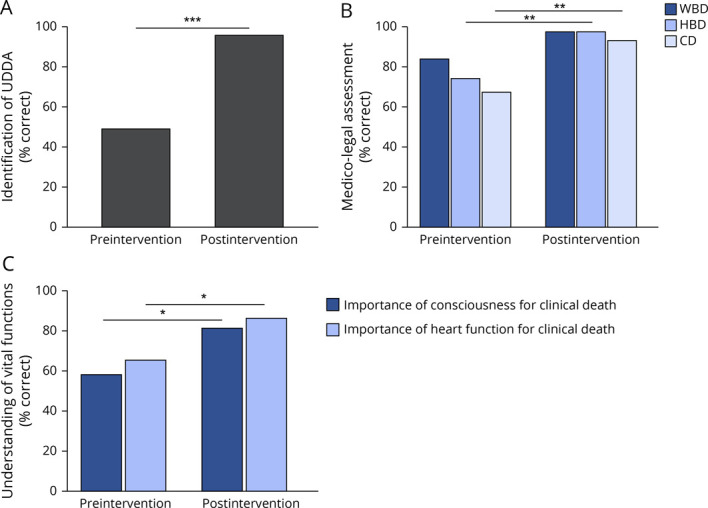
Medical Students Improved Their Understanding of the Medico-Legal Standard of Death (A) Students were more capable of selecting the criteria contained in the UDDA after the intervention. (B) Students improved in their assessment of whether a case of WBD, HBD, or CD is consistent with current medico-legal standards (C) Significantly more students understood that death can be declared while the heart is beating and that loss of consciousness does not entail death. Statistics were calculated using a McNemar test with bimodal distribution. *p < 0.05, **p < 0.01, and ***p < 0.001. CD = circulatory death; HBD = high-brain death; UDDA = Uniform Determination of Death Act; WBD = whole-brain death.

Following the educational intervention, students had a substantial increase in their understanding of the current medico-legal standards of death. More students (21%, OR 3.3, 95% CI 1.14–9.60) correctly indicated that patients can be declared dead while their heart is beating and that patients with irreversible unconsciousness, but preserved brain stem function, are not considered dead (23%, OR 3.15, 95% CI 1.18–8.38) (Figure 2A). There also was an increase in students who correctly identified a case of HBD (23%, OR 14.44, 95% CI 1.77–117.68) and CD (21%, OR 6.44, 95% CI 1.69–24.47) as inconsistent with current medico-legal standards (Figure 2B). The number of students who correctly answered all 3 vignettes also increased (40%, OR 7.96, 95% CI 2.63–24.1). However, there was no increase in students who correctly identified the WBD view as being consistent with current medico-legal standards (14%, OR 8.17, 95% CI 0.96–69.56). This is likely due to a ceiling effect whereby a high percentage (84%) of students identified the correct answer in the preintervention survey, thus limiting the amount of potential for increase. Students were also more likely to identify the correct criteria for death as described in the UDDA (46%, OR 21.48, 95% CI 4.6–100.19) (Figure 2C).

### Change in Attitude Toward 3 Views of Death Following Educational Intervention

**Figure 3:**
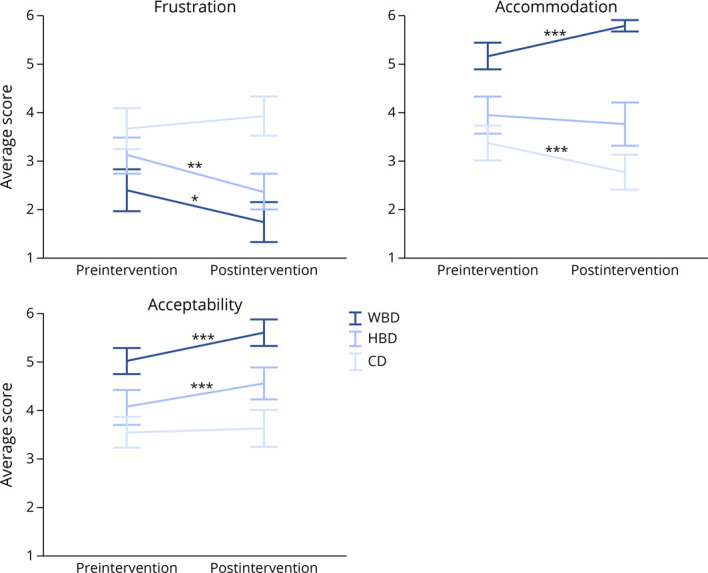
Differential Changes in Students' Attitudes Toward 3 Views of Death Change in average scores across the affective, behavioral, and cognitive domains of attitude for the 3 views of death. Responses were scored on a Likert scale from strongly disagree (1) to strongly agree (6). Error bars represent the SEM. Statistics represent difference in average score between preintervention and postintervention for each view of death. *p < 0.05, **p < 0.01, and ***p < 0.001. CD = circulatory death; HBD = high-brain death; WBD = whole-brain death.

After attending the intervention, students expressed less frustration toward the surrogate with a WBD view compared with before the lecture (${\bar{\text{χ}}}_{\text{diff}}$ = −0.64, 95% CI −0.15 to −1.15, *d* = 0.40). Students also described the surrogate's view as more acceptable (${\bar{\text{χ}}}_{\text{diff}}$ = 0.63, 95% CI 0.28–0.91, *d* = 0.87) and expressed a stronger likelihood of accommodating the surrogate's request to remove the ventilator (${\bar{\text{χ}}}_{\text{diff}}$ = 0.60, 95% CI 0.41–0.85, *d* = 0.59) after their attendance (Figure 3).

Similar results for the affective and cognitive measures were seen in the HBD scenario. Students expressed less frustration (${\bar{\text{χ}}}_{\text{diff}}$ = −0.74, 95% CI −0.28 to −1.21, *d* = 0.50) toward the surrogate with an HBD view and rated the surrogate's view as more acceptable after the lecture (${\bar{\text{χ}}}_{\text{diff}}$ = 0.52, 95% CI 0.25–0.80, *d* = 0.59). On average, students were no more likely to accommodate the surrogate's request of removing the ventilator after watching the lecture than they were before the lecture (${\bar{\text{χ}}}_{\text{diff}}$ = 0.19, 95% CI 0.67 to −0.30, *d* = 0.12) (Figure 3). Although an average shift was not identified, there was movement within the sample, with 13 students (30%) shifted toward a lesser likelihood of removing the ventilator and 16 (37%) shifted toward greater likelihood.

With respect to the CD view, students expressed comparable levels of frustration before and after the lecture (${\bar{\text{χ}}}_{\text{diff}}$ = −0.26, 95% CI 0.20 to −0.70, *d* = 0.17) (Figure 3). However, there was movement within the sample, where 10 students (23%) shifted toward less frustration and 19 (44%) shifted toward greater frustration. A similar pattern was seen in acceptability for the CD view. Although there was no global change from before the lecture to after the lecture (${\bar{\text{χ}}}_{\text{diff}}$ = −0.26, 95% CI −0.37 to 0.13, *d* = 0.15), 7 (16%) students shifted toward less acceptability, and 9 (21%) shifted toward greater acceptability. Students were less likely to accommodate the surrogate's request to continue ventilatory support in a patient with WBD after watching the lecture (${\bar{\text{χ}}}_{\text{diff}}$ = −0.61, 95% CI −0.91 to −0.30, *d* = 0.60) (Figure 3).

### Differential Effect on Students' Attitudes Toward 3 Views of Death Following Educational Intervention

Consistent with prelecture results, after attending the lecture, students expressed less frustration (${\bar{\text{χ}}}_{\text{diff}}$ = −0.63, 95% CI −1.03 to −0.22, *d* = 0.48), greater acceptance (${\bar{\text{χ}}}_{\text{diff}}$ = 1.05, 95% CI 0.63–1.46, *d* = 0.79), and a higher likelihood of accommodation (${\bar{\text{χ}}}_{\text{diff}}$ = 2.02, 95% CI 1.56–2.48, *d* = 1.36) for the WBD view compared with the HBD view (Table). Patterns of differences from preintervention to postintervention in frustration and acceptability were comparable between the WBD and HBD scenarios (Figure 3), but there was a greater difference toward greater likelihood of accommodation (${\bar{\text{χ}}}_{\text{diff}}$ = 1.39, 95% CI 0.39–2.39, *d* = 0.69) for the WBD surrogate compared with the HBD surrogate (Figure 3). Consistent with the prelecture results, students expressed less frustration (${\bar{\text{χ}}}_{\text{diff}}$ = −2.19, 95% CI −2.76 to −1.61, *d* = 1.16), more acceptance (${\bar{\text{χ}}}_{\text{diff}}$ = 1.98, 95% CI 1.44–2.52, *d* = 1.14), and higher likelihood of accommodation (${\bar{\text{χ}}}_{\text{diff}}$ = 3.02, 95% CI 2.60–3.45, *d* = 2.18) for the WBD surrogate compared with the CD surrogate after watching the lecture (Table). Although there were no differences in attitudes between the HB and CD view before the lecture, afterward, students expressed less frustration (${\bar{\text{χ}}}_{\text{diff}}$ = −1.56, 95% CI −2.11 to −1.00, *d* = 0.86), more acceptance (${\bar{\text{χ}}}_{\text{diff}}$ = 0.92, 95% CI 0.43–1.43, *d* = 0.58), and greater accommodation (${\bar{\text{χ}}}_{\text{diff}}$ = 1.00, 95% CI 0.37–1.63, *d* = 0.49) for the HBD view compared with the CD view (Table).

**Table:** Scores for Level of Frustration, Likelihood of Accommodating the Surrogate's Request, and Acceptability for the 3 Views of Death Before and After Attending the Educational Intervention

Attitude measure	View of death	Mean score	SEM	*p* Value		
				WBD and HBD	WBD and CD	HBD and CD
Preintervention						
Frustration	WBD	2.40	0.21	<0.01		
	HBD	3.12	0.19		<0.001	
	CD	3.67	0.21			0.07
Accommodation	WBD	5.16	0.13	<0.001		
	HBD	3.95	0.19		<0.001	
	CD	3.37	0.18			0.07
Acceptability	WBD	5.02	0.13	<0.001		
	HBD	4.07	0.18		<0.001	
	CD	3.56	0.16			0.06
Postintervention						
Frustration	WBD	1.74	0.21	<0.01		
	HBD	2.37	0.18		<0.001	
	CD	3.93	0.20			<0.001
Accommodation	WBD	5.79	0.06	<0.001		
	HBD	3.77	0.23		<0.001	
	CD	2.77	0.18			<0.01
Acceptability	WBD	5.62	0.14	<0.001		
	HBD	4.57	0.17		<0.001	
	CD	3.64	0.19			<0.001

Abbreviations: CD = circulatory death; HBD = high-brain death; WBD = whole-brain death.

Statistics were calculated using a paired *t* test and represent the difference in means.

## Discussion

In 1981, the UDDA was created to standardize the definition of death. Despite its widespread adoption, there remains active debate on whether the UDDA is an appropriate statutory definition of death.^17-19^ Medical students tend to have a poor knowledge of the criteria of brain death diagnosis,^5,6,8^ yet little investigation has gone into students' attitudes toward different conceptions of death.

Consistent with previous research, our study identifies deficits in medical student knowledge regarding the current medico-legal standards of death. Less than half of medical students could correctly identify the criteria contained in the UDDA before attending the lecture. Media has been reported to be a main source of information on brain death for medical students.^5^ Unfortunately, clinical death is often misrepresented by the use of confusing language such as life support in the context of brain death.^20-22^ Inaccurate reporting may negatively influence medical students' understanding of the basic facts of brain death. Furthermore, students performed better when asked if a case of WBD, HBD, or CD is consistent with the UDDA than they did when asked to select the criteria contained in the statute. Although both questions were intended to gauge baseline knowledge of the UDDA, students' improved performance in the case-based vs the text-based question underscores the importance of case-based learning in brain death education. Case-based applications appear especially effective for fleshing out nuanced ethical questions surrounding death determination.

Although there was a positive baseline attitude toward the high-brain view of death, the whole-brain view of death garnered the greatest acceptance and likelihood of ventilator removal. This may suggest that preserved brain stem function has a significant effect on students' intuitive attitude toward death. The different attitudes between the HBD and WBD views mirror contemporary debate surrounding death, with brain stem function perceived as morally significant, namely because of its implications for anencephalic infants and those in a chronic vegetative state.^23^

Of interest, independent of whether the students were correct or incorrect in their legal assessment of each view of death, attitudes were more positive for those views the students perceived to be the medico-legal standard. This likely owes to a confirmation bias among students, where their intuition for the medico-legal standing of the view informs their attitudes toward that view. The relationship between student perceptions of the medico-legal standard and their attitudes toward that perceived standard suggests that students may have attitudes that are based on false assumptions. This highlights the importance of interventions centered on clarifying the contemporary medico-legal standards of death.

Our findings of less positive attitudes toward CD suggest that second-year medical students hold neurocentric views of death. The increased acceptance and decreased frustration for the WBD and HBD views after the intervention were not seen for CD. In the qualitative responses, many students appealed to the irreversible loss of consciousness as an appropriate line for the end of life in the WBD and HBD scenarios. In the prelecture responses, these opinions came from their own personal view of death, whereas in the postlecture responses, while some responses still included statements on personal views of death, the majority included reference to the UDDA or medico-legal standards as justification for having a whole-brain view of death. After the lecture, many students noted that they would consider a patient with irreversible loss of consciousness with preserved brain stem function dead despite accurately identifying that this view is inconsistent with current medico-legal standards. This observation is in line with some bioethicists who argue for a revision to the UDDA to include a greater focus on capacity for consciousness.^16^ Furthermore, our data suggest that students could experience significant moral distress when advanced measures are performed on a patient in a persistent vegetative state who they personally view as dead.

The circulatory view of death did not garner the same overall increase in positive attitude after attending the lecture as did the high-brain view, but there was, nevertheless, significant individual change in attitudes. This may reflect the fact that this was the only case that asked the students to continue ventilator support instead of removing it, which, for some students, introduced other kinds of reasons to accept or refuse the surrogate's request. However, another explanation was the presence of consequentialist vs deontological frameworks for analyzing death. These 2 frameworks were present in the open-ended responses, where some students exhibited a death consequentialist perspective, opposing the continuation of ventilatory support in light of concerns about resource strain and allocation. For example, one student remarked that “it seems a waste of resources to continue this indefinitely when there is irreversible cessation of the function of the entire brain,” while another considered organ donation in the context of brain death, “the patient is brain dead, organs should be donated.” Conversely, other students exhibited a death deontologist perspective, noting the historical primacy of the circulatory view while recognizing the difficulty the spouse may experience in accepting the diagnosis of death by neurologic criteria. For example, one student noted, “I don't feel comfortable saying that their view of death is unacceptable, since there are varying views on this topic, many of which are rooted in religion or other deep-seated beliefs.” Similarly, another wrote, “although this is not in accordance with the UDDA guidelines I do think that it is fairly commonplace for someone to equate heart beat to life. So, for this reason I would not be as frustrated with the surrogate's view of life.”

Students who shifted toward greater acceptance and less frustration toward the surrogate with the CD view were more likely to be death deontologists, analyzing the circulatory view on more principled grounds (e.g., respect, tolerance, and autonomy), and often including words of sympathy or understanding for the surrogate in their open-ended responses. Conversely, the students who shifted toward less acceptance and greater frustration were more likely to be death consequentialists, analyzing the circulatory view in reference to how it operates in the larger scheme of medicine, and including references to distributive justice and futility of treatment. Both approaches contain merit. A deontological analysis of death promotes greater empathy for nonstandard views, whereas a consequentialist analysis pushes toward the current medico-legal standard.

Responses to a surrogate's request to remove a ventilator from a patient in a chronic vegetative state yielded 3 roughly equal subsamples (increased likelihood, decreased likelihood, and no change in likelihood). In this case, the vignette notes that the surrogate views irreversible loss of higher brain function as constituting death and requests the removal of the ventilator. Although there may be other kinds of ethical justifications for removal of the ventilator, none of those are specified in this case. Thus, because this patient does not meet the criteria for death under the UDDA, death cannot be used as the reason for ventilator removal.

The variation in the narrative responses to the HBD case reflects the broader ethical complexity of treatment decisions for patients with HBD. Students who reported being less likely to withdraw the ventilator interpreted the question in the context of the UDDA. For example, one student wrote, “I would want to follow the surrogate's request, but I know the patient has brainstem function so I don't know if it would be allowed because the current standards say that brain death needs to have complete stop of function.” Another noted, “This case would be difficult because though I completely understand the surrogate's view, UDDA doesn't agree and therefore I cannot take her and her spouse's view of life/death into account in determining to withdraw care.” Conversely, the students who were more likely to remove the ventilator tended to set the UDDA aside in their reasoning, saying things like “I agree with surrogate, as long as there's no chance the patient would wake up” and “I think that it is acceptable for the family member to withdraw care at this time because of quality-of-life issues.” Both interpretations of the vignette are correct in their own ways. Students who centered their response in the UDDA properly identified that HBD is not the current medico-legal standard of death, and therefore, viewing the patient as dead is an impermissible reason for removing the ventilator. Alternatively, students who set the UDDA aside in their answers posited reasons a surrogate might give when deciding to remove the ventilator for reasons of substituted judgment or best interest. These responses suggest that educators should place special emphasis on distinguishing the UDDA and its attendant standards from other ethical principles or positions that might nonetheless guide a treatment decision, particularly in cases of patients in a chronic vegetative state for whom decision making can be complex and lead to divergent outcomes.

The present study demonstrates the insufficiency of brain death knowledge among medical students and the importance of early educational interventions in bridging this gap. Despite the positive influence of the intervention, it was not without limitations. The effectiveness of the training was limited by the number of students participating in the in-person session. Small group discussions were not possible for students who chose to view the lecture recording, which may have attenuated their reflection on the concepts. A small sample size of 43 limits the generalizability of the study. Implementation of the intervention with subsequent classes of students would help clarify the effect of the intervention. Furthermore, the depth and breadth of the debate surrounding the definition of death puts a limit what can be taught in the span of 80 minutes. The intervention contained a comprehensive, but not exhaustive, review of the history, application, and debate surrounding death. The complex nuances of law, medicine, and ethics that exist in the context of clinical death are ever evolving. Thus, development of a longitudinal curriculum on the philosophy of death is warranted to more fully capture the multitude of complexities that are inherent in this issue. Long-term follow-up would help elucidate the durability of such interventions on influencing student knowledge and attitudes. Future studies should investigate other emotions, attitudes, and behaviors of medical students during situations of end-of-life care with surrogate decision makers who endorse nonstandard views of death. We did not control for prior exposure to brain death in this study. Furthermore, brain death is a rare occurrence, and considering that the cohort was still in their preclinical curriculum, we believed that prior exposure to brain death is unlikely to confound the analyses. However, prior experience with brain death may alter both students' knowledge and attitudes toward it and other nonstandard views of death. Investigation into students' prior experience with brain death and its effect on their knowledge and attitudes toward medico-legal standards would highlight the importance of lived experience in medical education. Education on brain death, its history and application, and varying philosophies of death are imperative for creating a future generation of physicians who can practice with a greater understanding of the UDDA and foster improved attitudes toward surrogates with nonstandard views of death that are more conducive to compassionate care.

## Appendix Authors

**Table:**

Name	Location	Contribution
Nicholas Ludka, BA	Department of Foundational Medical Studies, Oakland University William Beaumont School of Medicine, Rochester, MI	Drafting/revision of the manuscript for content, including medical writing for content; major role in the acquisition of data; study concept or design; and analysis or interpretation of data
Abram Brummett, PhD, HEC-C	Department of Foundational Medical Studies, Oakland University William Beaumont School of Medicine, Rochester, MI	Drafting/revision of the manuscript for content, including medical writing for content; major role in the acquisition of data; study concept or design; and analysis or interpretation of data
Jason Adam Wasserman, PhD, HEC-C	Department of Foundational Medical Studies, Oakland University William Beaumont School of Medicine, Rochester, MI	Drafting/revision of the manuscript for content, including medical writing for content; study concept or design; and analysis or interpretation of data

## Glossary

CD: circulatory death
HBD: high-brain death
OR: odds ratio
UDDA: Uniform Determination of Death Act
WBD: whole-brain death
